# BCR and its mutants, the reciprocal t(9;22)-associated ABL/BCR fusion proteins, differentially regulate the cytoskeleton and cell motility

**DOI:** 10.1186/1471-2407-6-262

**Published:** 2006-11-07

**Authors:** Xiaomin Zheng, Saskia Güller, Tim Beissert, Elena Puccetti, Martin Ruthardt

**Affiliations:** 1 Laboratory for Tumor Stem Cell Biology, Department of Hematology, J.W. Goethe University, Frankfurt, Germany

## Abstract

**Background:**

The reciprocal (9;22) translocation fuses the *bcr *(breakpoint cluster region) gene on chromosome 22 to the *abl *(Abelson-leukemia-virus) gene on chromosome 9. Depending on the breakpoint on chromosome 22 (the Philadelphia chromosome – Ph+) the derivative 9+ encodes either the p40^(ABL/BCR) ^fusion transcript, detectable in about 65% patients suffering from chronic myeloid leukemia, or the p96^(ABL/BCR) ^fusion transcript, detectable in 100% of Ph+ acute lymphatic leukemia patients. The ABL/BCRs are N-terminally truncated BCR mutants. The fact that BCR contains Rho-GEF and Rac-GAP functions strongly suggest an important role in cytoskeleton modeling by regulating the activity of Rho-like GTPases, such as Rho, Rac and cdc42. We, therefore, compared the function of the ABL/BCR proteins with that of wild-type BCR.

**Methods:**

We investigated the effects of BCR and ABL/BCRs i.) on the activation status of Rho, Rac and cdc42 in GTPase-activation assays; ii.) on the actin cytoskeleton by direct immunofluorescence; and iii) on cell motility by studying migration into a three-dimensional stroma spheroid model, adhesion on an endothelial cell layer under shear stress in a flow chamber model, and chemotaxis and endothelial transmigration in a transwell model with an SDF-1α gradient.

**Results:**

Here we show that both ABL/BCRs lost fundamental functional features of BCR regarding the regulation of small Rho-like GTPases with negative consequences on cell motility, in particular on the capacity to adhere to endothelial cells.

**Conclusion:**

Our data presented here describe for the first time an analysis of the biological function of the reciprocal t(9;22) ABL/BCR fusion proteins in comparison to their physiological counterpart BCR.

## Background

The t(9;22)(q34;q11) is detected in 95% of CML and 20–30% of adult ALL. CML is a myeloproliferative syndrome [1]. In contrast, Ph+-ALL is an acute disease characterized by blasts blocked at the pre-lymphatic stage of differentiation. Patients suffering from Ph+-ALL constitute a high risk group of ALL (5–10% survival rate after five years)[2]. The factors determining the biological differences between CML and Ph+-ALL are completely unknown.

The t(9;22) is a reciprocal translocation. A portion of chromosome 9 translocates onto chromosome 22 (22+), thereby replacing a fragment which in turn translocates onto chromosome 9 (9+) [1]. The derivative of chromosome 22 (22q+) can be revealed by cytogenetic techniques as the so-called Philadelphia chromosome (Ph).

On chromosome 22, translocation (9;22) involves the *bcr *(breakpoint cluster region) locus and there are two principal regions in which the breaks occur: (major) M-bcr, which spans between exons 12 to 16, and (minor) m-bcr, in the first intron, about 50 kb 5' of M-bcr. The product of fusion between M-bcr and abl is a protein of 210 kDa, the p210^((BCR-ABL))^, which is highly specific for CML. Due to the fact that the m-bcr maps within an intron, the p185^((BCR-ABL)) ^transcript in Ph+-ALL is constant [1]. Through fusion to BCR, the kinase activity of ABL becomes constitutively activated, leading to the constitutive activation of the "down-stream" signal transduction pathways, such as RAS, JAK-STAT and PI-3 kinase, responsible for the oncogenic potential of BCR/ABL [1]. The suppression of constitutively active ABL kinase by specific kinase inhibitors, such as Imatinib [3], Nilotinib [4] and Dasatinib. [5], reverts the oncogenic potential of BCR/ABL and these drugs are currently in clinical evaluation.

The breakpoint on chromosome 9 is located in intron 1 of the *abl *gene locus. It is, in contrast to the breakpoints on chromosome 22, constant and located between exons 1 and 2. The *abl/bcr *fusion genes on 9+ differ depending on the breakpoint on chromosome 22. Fusion between M-bcr and *abl *results in the 'small' *abl/bcr *fusion gene encoding a 'small' ABL/BCR transcript, detectable in 65% patients suffering from CML [6], which is translated into an ABL/BCR protein with a theoretical molecular mass of about 40 kDa – p40^(ABL/BCR) ^(Zheng et al. in preparation). The fusion between m-bcr and *abl *leads to a 'large' transcript, present in 100% of examined patients with a Ph+-ALL [7], which encodes a fusion protein with a theoretical molecular mass of about 96 kDa – p96^(ABL/BCR)^(Zheng et al. in preparation).

**Figure 1:**
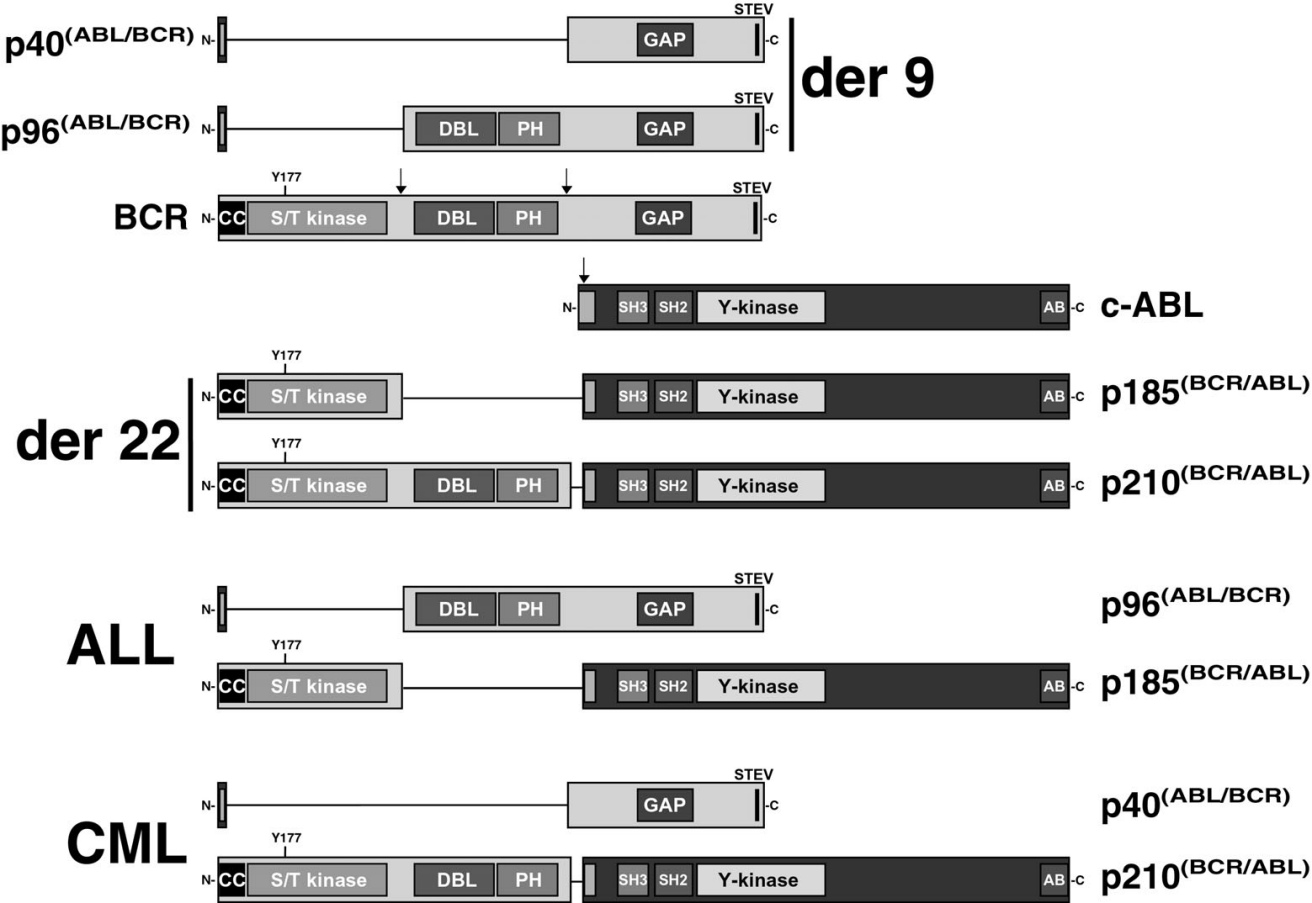
**Modular organization of the main t(9;22) translocation products**. The protein sizes are not to scale. CC – coiled coil oligomerization interface; S/T kinase – serine/threonine kinase domain; DH – dbl homology domain; PH – pleckstrin homology domain; GAP – GTPase activation domain; STEV – PDZ-binding motif; SH3, SH2 – Src-homology domain 3 and 2; Y-kinase – tyrosine kinase domain (SH1), AB – actin-binding domain; der 9 and der 22 – derivative 9 and 22.

The ABL/BCR fusion proteins represent mutants of the protein kinase BCR. BCR is a Rho-GEF due to the presence of a dbl homology (DH) domain and a pleckstrin homology (PH) domain (Fig. 1) [8,9]. The GEFs activate members of the Ras superfamily by increasing the proportion of their GTP-bound form with respect to the GDP-bound form [10]. The prototype for Rho-GEF, the diffuse B-cell lymphoma (Dbl) oncogene, has a strong transformation activity in NIH3T3 fibroblasts, and both the Rho-GEF function and the oncogenic potential of Dbl depend on its DH-domains [11].

Furthermore, BCR also contains a C-terminal Rac-GAP domain. In contrast to GEFs, the GTPase activating proteins (GAPs) promote hydrolysis of GTP to GDP and increase the inactive forms of small GTPases of the Ras superfamily [10]. In fact, BCR is a negative regulator of Rac, as demonstrated by the fact that it reduces the Rac1-dependent activation of the protein kinase Pak1, an activator of the JNK pathway, via its GAP function [12].

The Rho-GEF and Rac-GAP functions strongly suggest an important role of BCR in cytoskeleton modeling by regulating the activity of Rho-like GTP-ases, such as Rac, cdc42, and Rho [11]. Rho, cdc42 and Rac are involved in the formation and maintenance of 'stress fibers', filopodia and lamellipodia, respectively [13]. The N-terminal 'coiled coil' dimerization interface of BCR, its serine/threonine kinase activity, and the tyrosine phosphorylation site at position 177 (Y177) are indispensable for its function [1,14].

Both p40^(ABL/BCR) ^and p96^(ABL/BCR) ^are mutated BCR, but nothing is known about their biology. In the ABL/BCR fusion proteins, the N-terminus of BCR is substituted by the first exon of *abl*. The differences between ALL- and CML-specific ABL/BCR lie in the presence of the DH and PH domains. CML-associated p40^(ABL/BCR) ^lacks the DH/PH domains conserved in the ALL-specific p96^(ABL/BCR) ^(Fig. 1). Thus the ALL-specific p96^(ABL/BCR) ^fusion protein is an N-terminally truncated Rho-GEF and, therefore, a putative oncogene [15,16].

To determine the role of the ABL/BCR in Ph+ leukemia we compared the effects of BCR and the ABL/BCR proteins on the regulation of Rho-like GTPases, cytoskeleton modeling and cell motility.

## Methods

### Cell lines and Ph+ leukemic blast culture

Cells were cultured at 37°C in 5% CO2 and a humidified atmosphere. Rat-1 cells and the ecotropic packaging Phoenix cell line were maintained in DMEM (Invitrogen, Karlsruhe, Germany) supplemented with 10% FCS (Hyclone, Perbio, France). 32D cells were maintained in RPMI 1640 medium (Invitrogen) supplemented with 10% FCS (Invitrogen) and 10 ng/ml mouse IL-3 (Cell Concepts, Umkirch, Germany). The murine bone marrow stroma cell line M2-10B4 (kindly provided by Gesine Bug, Experimental Hematology, J.W. Goethe University) was maintained in RPMI 1640 (Invitrogen) with 10% FCS (Hyclone), 0.06 mg/ml hygromycin B (Merck/Calbiochem, Schwalbach, Germany), 0.4 mg/ml geneticin and 8 mM HEPES (Invitrogen).

### Western blotting

Western blot analysis was performed according to widely used protocols using antibodies directed against ABL (α-ABL)(Santa Cruz Biotechnology, Santa Cruz, California, USA), BCR (α-BCR)(Santa Cruz), cdc42 (α-cdc42)(Pierce, Bonn, Germany), Rac (α-Rac) (Pierce) and Rho (α-Rho) (Santa Cruz). Blocking was performed in 5% low fat dry milk (α-ABL), 3% bovine serum albumin (BSA) (α-cdc42, α-Rac, α-Rho), Tris-buffered saline (TBS) containing 0.1% Tween 20 (TBS-T)(α-BCR). Washing was carried out in TBS-T. Antibody incubations were performed in either 3% BSA (α-cdc42, α-Rac, α-Rho) or TBS-T (α-BCR). Horseradish peroxidase-conjugated secondary antibodies were diluted 1:2000 in 0.5% low-fat dry milk and chemiluminescence was revealed by autoradiography.

### Plasmids

The cDNAs encoding p40^(ABL/BCR) ^and p96^(ABL/BCR) ^were cloned from the BV173 and SupB15 cell lines respectively by RT-PCR (ABL-ATG 5'- GCA AAA TGT TGGAGA TCT GCC TG -3'; BCR rev 5'- CTC GTA GAG CTC AGG CAC TTT G -3') and confirmed by sequencing. Plasmids containing the activated forms of Rac (V12Rac), cdc42 (V12cdc42) and Rho (V14Rho) were kindly provided by P. G. Pelicci, IEO, Milan Italy. All constructs were subcloned into pENTR.1A ("Gateway" recombination system – Invitrogen) for further transfer into expression vectors previously converted to Gateway destination vectors according to the manufacturer's instructions (Invitrogen). For retroviral transduction we used retroviral vectors based on the PINCO vector, which harbors the enhanced green fluorescence protein (EGFP) as reporter under the control of the cytomegalovirus promoter [17].

### Retroviral transduction

Phoenix cells were transfected with retroviral vectors by calcium phosphate precipitation as described before [17]. Retroviral supernatant was collected at days 2 and 3 after transfection. Target cells were plated onto Retronectin-coated (Takara-Shuzo, Shiga, Japan) non-tissue culture-treated 24-well plates. They were exposed to the retroviral supernatant and then centrifuged at 600 g for 45 minutes. After incubation for another 3 hours at 37°C, the retroviral supernatant was replaced. Infection was repeated 4 times and transduction efficiency had to be at least 70%, as assessed by the detection of EGFP-positive cells by fluorescence-activated cell sorting (FACS). Differences in transduction efficiency between the samples did not exceed 10%.

### Rho-, Rac- and cdc42-activation assays

Rho-, Rac- and cdc42-activation assays were performed with the GTPase activation kit (Pierce) according to the manufacturer's instructions. After retroviral transduction, Rat-1 cells were grown to 90–100% confluence in 100 mm culture dishes and lysed in 500 μl lysis/binding/washing buffer (25 mM TrisHCl, pH 7.5, 150 mM NaCl, 5 mM MgCl2, 1% NP-40, 1 mM DTT, 5% glycerol). After centrifugation the clarified cell lysates (500 μg) were incubated with GST-Pak1-PBD (to pull down active cdc42 or Rac1) or GST-Rhotekin-RBD (to pull down active Rho) in the presence of SwellGel Immobilized Glutathione at 4°C for 1 hour in a spin column. After incubation, the mixture was centrifuged at 8,000 × g to remove the unbound proteins. The resins were washed three times with lysis/binding/washing buffer and the samples were eluted by adding 50 μl of 2X SDS sample buffer and boiling at 95°C for 5 minutes. Half (25 μl) of the sample volumes were analyzed by SDS-PAGE and transferred to a nitrocellulose membrane. Active Ras, cdc42, Rac1 or Rho were detected by Western blotting using specific antibodies.

### Immunofluorescence

Rat-1 cells grown on coverslips were fixed with 2% paraformaldehyde in phosphate-buffered saline (PBS) for 15 min, washed twice with PBS and then permeabilized with 0.1% Triton X-100 for 20 min. The cells were then blocked with 2% BSA for 20 min. For actin staining, the cells were incubated with Cy3-conjugated phalloidin (Invitrogen) for 15 min at RT. The stained cells were mounted onto slides in moviol and pictures were taken with an Axioplan II microscope and digitalized by the Axiovision software (Zeiss, Göttingen, Germany).

### Spheroid assay

M2-10B4 spheroids were grown in 1% agarose-coated 96-well plates using the liquid overlay technique. For initiation, 2.5 × 10^4 ^cells were inoculated per well in 200 μl Iscove's modified Dulbecco's medium (IMDM; Biochrom) supplemented with 10% FCS. After 4 days, 1 × 10^4 ^infected 32D cells were added for cocultivation by replacing 100 μl of the medium. Spheroid cocultures were harvested 6–96 h later, washed with PBS and dissociated by incubation with a 0.25% trypsin and 0.1% EDTA solution (1:3 in PBS; PAN Biotech, Aidenbach, Germany) for 5 min at 37°C and mechanic pipetting. Cell suspensions were filtered through a mesh (pore size 70 μm; Falcon, Becton Dickinson Labware, Le Pont de Claix, France) and EGFP was measured by FACS to determine the percentage of 32D cells in the spheroids. Analysis was performed on a FACScan (BD Bioscience) using CellQuest software.

### Adhesion assay under shear stress – flow chamber

The apparatus consisted of a circular flow chamber with an inlet port through which the 32D cells were injected, an outlet port, where the flow-out medium and cells passed through, and a vacuum port which created a vacuum. This apparatus was mounted on a glass slide coated with an endothelial layer (HUVEC). The entire setup was placed on an inverted microscope connected to a video system to document the motility of the cells. The 32D cells were flushed into the chamber with a constant shear stress (0.1 and 2 dyn/cm^2^) through a tubing system connected to an injection syringe, which in turn was operated by a perfusion pump with a velocity that could be modified manually (2.5 to 99 ml/hr). The system created a laminar flow of liquid over the endothelial layer. Initially, the cells were flushed at a constant shear stress of 0.1 dyn/cm^2 ^(2.5 ml/h) at a rate of ~4 × 10^4 ^cells/min for 10 minutes over the endothelial layer. Representative pictures of three different fields were taken to assess the adhesion of cells under low shear stress. The shear stress was increased to a rate of 2 dyn/cm^2 ^for an additional 10 min to flush away weakly bound cells.

### Endothelial transmigration

The endothelial transmigration assay was performed using transwells with a pore size of 5 μm (BD-Falcon, Heidelberg, Germany). HUVEC were seeded onto 0.1% gelatine-coated transwells at a concentration of 30.000 cells/well. The non-adherent cells were removed 24 hours after seeding by flushing with medium. The confluence and the membrane integrity of the endothelial cells were determined by measuring the permeability for fluorescein isothiocyanate (FITC)-dextran 3000. Endothelial cells were washed twice with the assay medium (DMEM containing 0.1% (w/v) BSA) before 25.000 32D cells were added to each upper compartment with 0.3 ml assay medium. The plate was incubated at 37°C, 5% CO_2 _for 6 hours. The cells that migrated to the lower compartment were collected and mixed with 10^5 ^untransduced 32D cells. The transmigarting cells were then quantified by measuring the EGFP by FACS.

## Results

### Differential cytoskeleton modeling by p40^(ABL/BCR)^, p96^(ABL/BCR) ^and BCR

The reciprocal ABL/BCR fusion proteins are BCR mutants. BCR has Rho-GEF and Rac-GAP activity, strongly suggesting a main role in the regulation of cytoskeleton modeling and cell motility by modulating the activity of the Rho subfamily of small GTPases, such as Rac, cdc42 and Rho. The basis for this is that Rho-GTPases regulate actin filament assembly. To determine how the BCR mutations related to t(9;22) interfere with its functionality we compared the phenotypes induced by p40^(ABL/BCR) ^and p96^(ABL/BCR) ^with that of wt BCR.

**Figure 2:**
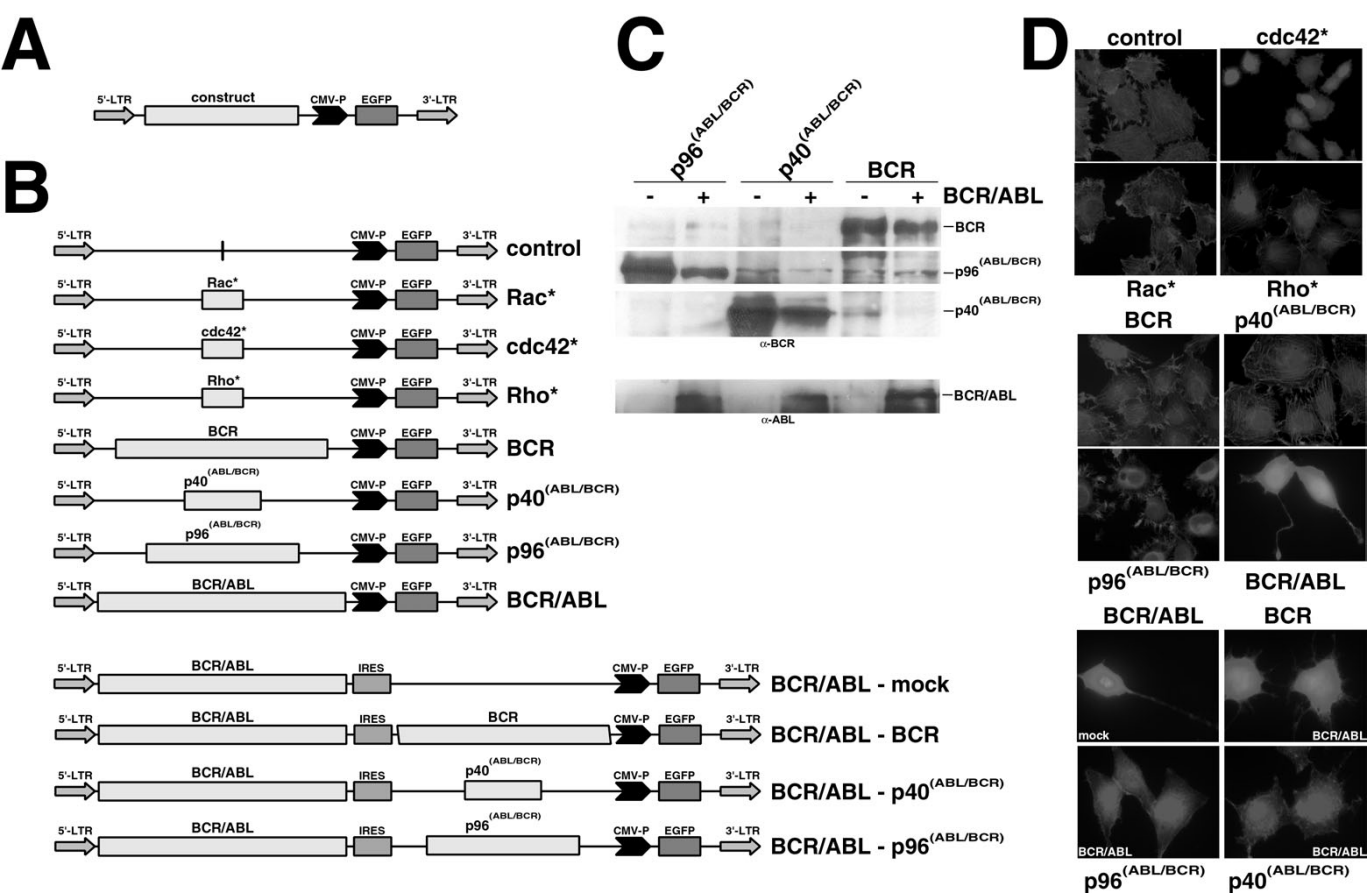
**Cytoskeleton modeling in Rat-1 cells expressing t(9;22) translocation products**. **A. **The provirus used for transduction of the cell line. Expression of the transgenes is driven by the 5'-LTR (long-term repeat), whereas the EGFP-reporter gene is under the control of the CMV promoter (CMV-P). **B. **Provirus used for the expression of the transgenes. The sizes are not to scale. **C. **Expression of the transgenes in Rat-1 cells was controlled by Western blot analysis using α-ABL and α-BCR antibodies as indicated. **D. **Rat-1 cells expressing the indicated transgenes stained with phalloidin to reveal the actin cytoskeleton. mock – empty vector infected cells as control. Control – mock infected Rat-1 cells; Rac* – V12Rac (constitutively activated form of Rac), cdc42* – V12cdc42 (constitutively activated form of cdc42), Rho* – V14Rho (constitutively activated form of Rho). The bottom panel shows the Rat-1 cells co-expressing BCR/ABL together with BCR or its mutants.

First, we investigated how the p40^(ABL/BCR) ^and the p96^(ABL/BCR) ^fusion proteins modulate the cytoskeleton in comparison to wt BCR. We used the bi-cistronic retroviral PINCO vector [17] for the expression of BCR, p40^(ABL/BCR)^, p96^(ABL/BCR) ^and p185^(BCR/ABL) ^in Rat-1 cells, with the protein of interest under the control of the long terminal repeat (LTR) and the EGFP driven by a CMV promoter (Fig. 2A). For controls, the cells were infected with the empty PINCO vector, able to express only EGFP (mock). All retroviral constructs used in these experiments are shown in Fig. 2B. Expression of the transgenes were controlled by Western blotting (Fig 2C). Cytoskeleton modeling was detected by labeling the actin filaments with Cy3-conjugated phalloidin. The over-expression of BCR led to a Rho-like phenotype characterized by stress fibers and microspikes (Fig. 2D). In contrast, p96^(ABL/BCR) ^induced a cdc42-like phenotype dominated by filopodia, whereas the p40^(ABL/BCR)^-related phenotype seemed to be a mixture between a Rac-like and a cdc42-like phenotype, due to the presence of both lamellopodia and filopodia (Fig. 2D). On the other hand, p185^(BCR/ABL) ^induced a phenotype characterized by complete polarization of the cell and the formation in a high number of cells of one long, axon-like filopodium and few shorter neurite-like filopodia, conferring a neuron-like aspect to the cells attributable to strong Rho-activation [13] as already seen in PC12 cells [18]. This phenotype was influenced by the co-expression of either BCR or the ABL/BCRs. Both BCR and p40^(ABL/BCR) ^abolished the polarization of the BCR/ABL-expressing cells which maintained the capacity to form the neurite-like filopodia. In contrast, BCR/ABL positive Rat-1 cells co-expressing p96^(ABL/BCR) ^maintained a polarized phenotype with lamellopodia.

In summary, these data suggest that the ABL/BCR fusion proteins are able to modify the cytoskeleton of fibroblasts differently to wt BCR and BCR/ABL. Furthermore it seems that there is a complex functional interaction between the t(9;22)-associated fusion proteins.

### Differential regulation of small Rho-like GTPases by p40^(ABL/BCR)^, p96^(ABL/BCR) ^and BCR

To determine the molecular mechanisms underlying the differences between BCR and its mutants regarding cytoskeleton modeling we looked for alterations in the regulation of small Rho-like GTPases. Therefore we assessed the activation status of endogenous Rho-like GTPases in Rat-1 cells expressing BCR and its mutants. As a control we used mock-transduced Rat-1 cells. Transduction efficiency was always more than 80%, as demonstrated by the percentage of EGFP-positive cells (data not shown).

**Figure 3:**
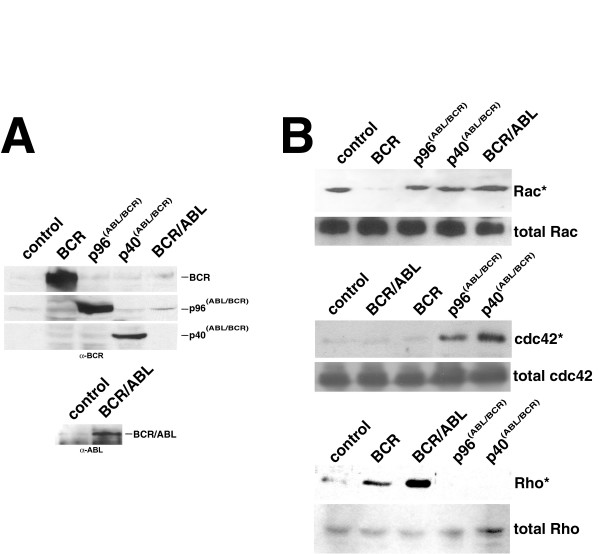
**Rho-like small GTPase activation in Rat-1 cells expressing t(9;22) translocation products**. **A. **Expression of the transgenes in Rat-1 cells was controlled by Western blot analysis using α-ABL and α-BCR antibodies as indicated. **B. **The indicated activated small GTPase was pulled down and revealed by the specific antibody. Rac*, cdc42*, Rho* – activated forms; total Rac, cdc42, Rho – total amount revealed in the cell lysates used for the pull down. One representative experiment of three performed is given.

As shown in Fig. 3, both p40^(ABL/BCR) ^and p96^(ABL/BCR) ^have lost the Rho-GEF function of BCR, because they were unable to activate Rho. The strong Rho-activation in BCR/ABL-expressing cells is most likely due to the ABL-kinase activity, given that the BCR/ABL fusion protein used in these assays lacks the Rho-GEF domain. Furthermore, both p40^(ABL/BCR) ^and p96^(ABL/BCR) ^have also lost the Rac-GAP function of BCR, as shown by their incapacity to suppress Rac-activation in Rat-1 cells (Fig. 3). In contrast, both have acquired the capacity to activate cdc42, whereas neither BCR nor BCR/ABL were able to activate cdc42 in these cells (Fig. 3)

Taken together, these data show that the ABL/BCR proteins present a pattern of small Rho GTPase activation which differs from that induced by wt BCR and BCR/ABL. In fact BCR can be considered a repressor of Rac and an activator of Rho that lacks any influence on cdc42, whereas the ABL/BCR proteins can be considered activators of cdc42 that have lost the capacity to activate Rho and to suppress Rac. In contrast, BCR/ABL is an activator of Rac and Rho, and has no influence on cdc42. These different activation patterns may account for the differences in cytoskeleton modeling.

### In contrast to wt BCR and p40^(ABL/BCR)^, p96^(ABL/BCR) ^inhibits and BCR/ABL increases the migration of hematopoietic progenitor cells in a three-dimensional stroma model

**Figure 4:**
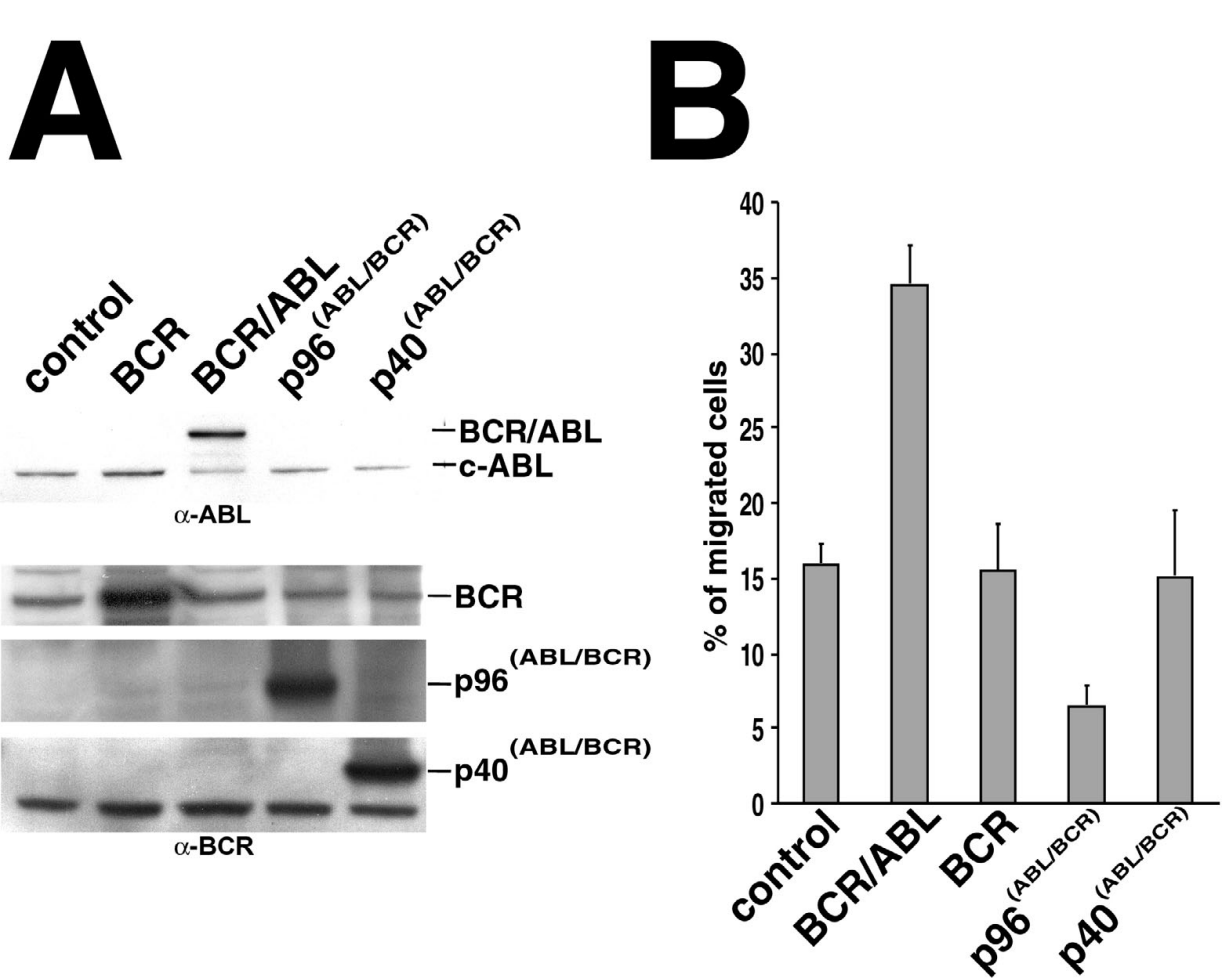
**Migration of hematopoietic progenitor 32D cells expressing t(9;22) translocation products into M2-10B4 spheroids**. **A. **Expression of the transgenes in 32D cells was controlled by Western blot analysis using α-ABL and α-BCR antibodies as indicated. **B. **Percentage of 32D cells expressing the indicated transgenes that migrated into the spheroids. The bar graphs represent the averages of triplicates with standard deviation. One representative experiment out of three performed is given.

Cytoskeleton modeling plays an important role in the biology of hematopoietic precursors because it contributes to cell motility, defined by migration, adhesion and chemotaxis. Motility is a main feature of hematopoietic stem cells and more mature progenitors, as well as of functional white blood cells. Therefore, we investigated the effect of retroviral expression of BCR, p40^(ABL/BCR)^, p96^(ABL/BCR) ^and BCR/ABL on the motility of the murine IL-3-dependent 32D myeloid precursor cell line. We used 32D cells, because of their capacity to migrate in the three-dimensional stroma spheroids. Transduction efficiency was always more than 70%, as assessed by the percentage of GFP-positive cells (data not shown) and expression of the transgenes was controlled by Western blotting (Fig. 4A).

The effects on migration were assessed in a three-dimensional *in vitro *spheroid model based on murine M2-10B4 bone marrow stroma cells. It reproduces the stromal microenvironment in the bone marrow [19] and allows to study the functional consequences of the complex interaction between cell membrane, adhesion systems and cell signaling pathways. It has been previously shown that migration into the M2-10B4 spheroids is closely related to the presence of activated Rho in the progenitor cells [19]. Here we show that BCR/ABL enhances the capacity of the 32D cells to migrate into the spheroids, whereas BCR did not increase migration. In contrast to p40^(ABL/BCR)^, which had no effect, the presence of p96^(ABL/BCR) ^inhibited the migration of 32D cells into the spheroids (Fig. 4B).

Taken together, these data provide evidence that BCR, ABL/BCR and BCR/ABL confer to 32D cells a different migration potential in the three dimensional spheroid model.

### In contrast to the ABL/BCR proteins, BCR increases and BCR/ABL diminishes the adhesion of hematopoietic progenitors to endothelium

**Figure 5:**
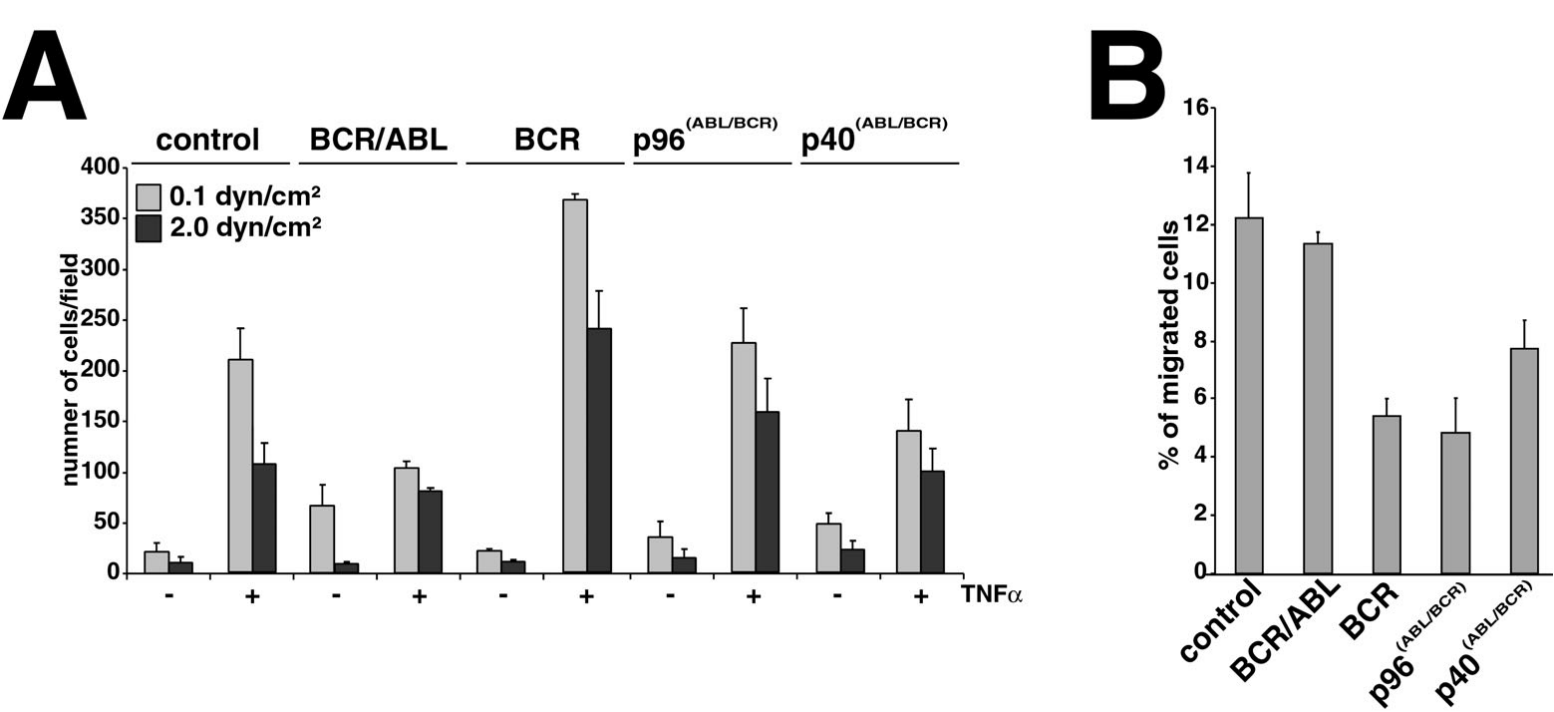
**Adhesion on endothelium and endothelial transmigration of hematopoietic progenitor 32D cells expressing t(9;22) translocation products**. **A. **Percentage of 32D cells that were adherent to HUVEC in the absence/presence (-/+) of TNFα under different shear stresses (0.1 and 2 dyn/cm^2^) in a flow chamber. The bar graphs represent the averages of three fields counted with standard deviation. One representative experiment out of three performed is given. **B. **Percentage of 32D cells that migrated through a HUVEC layer in a transwell assay. The bar graphs represent the average of triplicates with standard deviation. One representative experiment out of three performed is given.

Adhesion is a complex sequential process of capture, rolling and firm adhesion, mainly mediated by the P- and E-selectin on the endothelial cells and their binding proteins, such as PSGL-1, CD18, CD11a and Mac-1, on the leukocyte membrane. Adhesion can be modified by the activation pattern of Rho-like GTPases through an inside-out signaling mechanism [20]. Adhesion to endothelial cells under shear stress was assessed in a flow chamber model based on HUVEC cells. Here we investigated the capacity of 32D cells expressing wt BCR, p40^(ABL/BCR)^, p96^(ABL/BCR) ^or BCR/ABL to adhere to HUVEC cells in a flow chamber under different shear stress conditions (0.1 and 2 dyn/cm^2^). HUVEC cells were also stimulated by TNFα for the induction of selectin expression. The adhesion of the 32D cells to unstimulated HUVEC cells was not modified by the expression of the different transgenes, independently of shear stress. In contrast, BCR increased and p185^(BCR/ABL) ^reduced the adhesion of 32D cells on TNFα-stimulated HUVEC under both shear conditions (Fig. 5A). 32D cells expressing p40^(ABL/BCR) ^or p96^(ABL/BCR) ^did not show significant modifications in adhesion to TNFα-stimulated HUVEC with respect to controls (Fig. 5A). These modifications in adhesion were independent of the expression levels of PSGL-1 and Mac-1, because these were not modified by the transgenes, as determined by comparison with mock-transduced controls (data not shown).

### BCR and p96^(ABL/BCR) ^reduce endothelial transmigration in an SDF-1α gradient of hematopoietic precursors

Endothelial transmigration, which is mainly mediated by integrin expression on the endothelial cells, was investigated in a HUVEC-based transwell assay with an SDF-1α chemotactic gradient. BCR and p96^(ABL/BCR) ^reduced the chemotactic response of 32D cells, indicating a role of the Rho-GEF domain because BCR/ABL and p40^(ABL/BCR) ^did not influence transmigration in comparison to controls. These effects were independent of the integrin ligand proteins. In fact, Icam-1 expression was not modified in 32D cells by the transgenes (data not shown). Furthermore, the expression of the SDF-1α receptor CXCR4 on the surface of 32D cells was not modified by the expression of the transgenes (data not shown).

Taken together, these data indicate that the modification of the activation pattern of Rho-like GTPases by BCR, p185^(BCR/ABL) ^and the ABL/BCR fusion proteins, with the accompanying alterations in cytoskeleton modeling interferes with the motility of hematopoietic precursor cells.

## Discussion and Conclusion

BCR is not only a negative regulator of cell proliferation and oncogenic transformation [14], but plays moreover a crucial role in cytoskeleton modeling by regulating the activity of Rho-like GTPases, such as Rho, Rac and cdc42. Cytoskeleton modeling and cell motility are considered to be related [20].

Cell motility is a major feature of the functionality of hematopoietic cells, regardless of their differentiation stage. Cell motility regulates i.) the entrance and persistence of very early hematopoietic precursors into the bone marrow stroma; ii.) the mobilization of more mature white blood cells from the bone marrow into the peripheral blood; and iii.) the capacity of functional blood cells, such as granulocytes, macrophages, lymphocytes, to leave the blood stream and reach the areas where their function is required.

Deregulated cell motility, e.g. in leukemic precursors, can lead to i.) an abnormal mobilization of early leukemic precursors into the peripheral blood; ii.) a leukemic infiltration of extra-medullar tissues; and iii.) an abnormal capacity to pass through the blood-brain barrier with consequent meningeal leukemia, as frequently seen in Ph+-ALL [21].

The capacity of a cell to move is determined by the flexibility of its three dimensional architecture and thus by the regulation of the cytoskeleton modeling as well as by the capacity to interact dynamically with the support and the cellular environment by adhesion and migration. These features are not only dependent on the cell type but also on the stage of differentiation [22].

Here we show that the ability to deregulate cytoskeleton modeling and cell motility is not limited to the major t(9;22)-associated BCR/ABL, but is also exhibited by the reciprocal ABL/BCR fusion proteins.

The deletion in the ABL/BCR fusion proteins with respect to wt BCR leads to a deregulated activation pattern of Rho-like GTPases in Rat-1 cells. The BCR pattern of activated Rho, inhibited Rac and unmodified (as compared to the control) cdc42 is shifted to a ABL/BCR pattern of unmodified Rho and Rac, and activated cdc42. As the isolated Rho-GEF domain of BCR is a strong activator of cdc42 [23], the lack of cdc42 activation by full-length BCR and the strong activation by p96^(ABL/BCR) ^suggests the presence of a self-inhibitory domain in BCR which is deleted in p96^(ABL/BCR)^. Such an inhibitory domain could be located in the GAP domain, which alone is able to inhibit cdc42 [23]. A completely different but still unknown mechanism can be postulated following the observation that p40^(ABL/BCR) ^activates cdc42 although it lacks the GEF-domain.

The expression of BCR/ABL in Rat-1 cells led to a neuron-like phenotype probably related to very strong Rho-activation [13]. The pattern of activated Rho-like GTPases in BCR/ABL expressing Rat-1 cells differs from that in BCR/ABL-positive Ba/F3 cells, confirming the cell type specificity [24].

The t(9;22) associated fusion proteins do not only modify the cytoskeleton modeling but also the motility of cells as we have shown in the 32D hematopoietic precursor cell line. A relationship between cytoskeleton modeling and motility is suggested by the fact that migration into spheroid cells has been shown to be closely related to Rho-activation [19]. Although both BCR/ABL and BCR are Rho-activators in Rat-1 cells, BCR/ABL-positive cells showed an increased migration capacity as compared to BCR over-expressing cells. The inhibition of migration into the stromal spheroids by p96^(ABL/BCR) ^suggests an active mechanism, such as a dominant negative effect on endogenous BCR, whereas p40^(ABL/BCR) ^is probably unable to interfere with migration due to the absence of the Rho-GEF domain. Therefore it remains unclear whether there is a direct relationship between alterations of migration into the spheroids and altered cytoskeleton modeling.

The increased adhesion of BCR over-expressing 32D cells seems to be independent of the expression of selectin ligands expressed on the cell surface (data not shown) indicating another mechanism, which might not be activated by the ABL/BCR proteins. Our findings confirm recent data showing that BCR/ABL induces defects in integrin function in CML which could interfere with the capacity to adhere and thereby contribute to the dramatic accumulation of mature granulocytes in the peripheral blood [25]. In contrast to the adhesion process, both BCR and p96^(ABL/BCR) ^decreased endothelial transmigration, whereas BCR/ABL and p40^(ABL/BCR) ^had no influence. The effect of BCR/ABL seems to be cell type specific. In contrast to 32D cells BCR/ABL increases endothelial transmigration and chemotaxis of the lymphatic Ba/F3 cells [26]. The fact that adhesion is a part of the transmigration process [20] and that the expression level of the SDF-1α-receptor CXCR4 is not influenced by the transgenes (data not shown) strongly suggest that the differences are related to alterations in the leukocyte-endothelium interactions. The way in which BCR and p96^(ABL/BCR) ^interfere with the leukocyte endothelium interaction remains to be clarified. This is of high significance because it might influence the biology of Ph+ leukemia, as suggested by findings which show that the lack of the adhesion molecules P-selectin and Icam-1 accelerates the development of BCR/ABL-induced CML-like disease in mice [27].

Taken together, our data presented here show that the reciprocal t(9;22) ABL/BCR fusion proteins both lose functions of the physiological counterpart BCR and gain functions which BCR does not have. These new functionalities might influence the biology of Ph+ leukemia. The next step is to determine how BCR/ABL and the ABL/BCR proteins interfere with each other. Together with the data presented here, this will foster the understanding of the pathogenesis, clinical behavior and probably the response to therapy of Ph+ leukemia.

## Competing interests

The author(s) declare that they have no competing interests.

## Authors' contributions

XZ carried out the cell motility experiments.

SG produced the cell lines and performed the analysis of the cytoskeleton modeling.

TB carried out the pull-down assays.

EP participated in the design of the study and carried out data analysis

MR designed the study and wrote the paper

All authors read and approved the final manuscript

## Pre-publication history

The pre-publication history for this paper can be accessed here:



## References

[B1] Faderl S, Talpaz M, Estrov Z, S OB, Kurzrock R, Kantarjian HM (1999). The biology of chronic myeloid leukemia. N Engl J Med.

[B2] Gokbuget N, Hoelzer D, Arnold R, Bohme A, Bartram CR, Freund M, Ganser A, Kneba M, Langer W, Lipp T, Ludwig WD, Maschmeyer G, Rieder H, Thiel E, Weiss A, Messerer D (2000). Treatment of Adult ALL according to protocols of the German Multicenter Study Group for Adult ALL (GMALL). Hematol Oncol Clin North Am.

[B3] Deininger M, Buchdunger E, Druker BJ (2005). The development of imatinib as a therapeutic agent for chronic myeloid leukemia. Blood.

[B4] Kantarjian H, Giles F, Wunderle L, Bhalla K, O'Brien S, Wassmann B, Tanaka C, Manley P, Rae P, Mietlowski W, Bochinski K, Hochhaus A, Griffin JD, Hoelzer D, Albitar M, Dugan M, Cortes J, Alland L, Ottmann OG (2006). Nilotinib in imatinib-resistant CML and Philadelphia chromosome-positive ALL. N Engl J Med.

[B5] Tokarski JS, Newitt JA, Chang CY, Cheng JD, Wittekind M, Kiefer SE, Kish K, Lee FY, Borzillerri R, Lombardo LJ, Xie D, Zhang Y, Klei HE (2006). The structure of Dasatinib (BMS-354825) bound to activated ABL kinase domain elucidates its inhibitory activity against imatinib-resistant ABL mutants. Cancer Res.

[B6] Melo JV, Gordon DE, Cross NC, Goldman JM (1993). The ABL-BCR fusion gene is expressed in chronic myeloid leukemia. Blood.

[B7] Melo JV, Gordon DE, Tuszynski A, Dhut S, Young BD, Goldman JM (1993). Expression of the ABL-BCR fusion gene in Philadelphia-positive acute lymphoblastic leukemia. Blood.

[B8] Maru Y, Witte ON (1991). The BCR gene encodes a novel serine/threonine kinase activity within a single exon. Cell.

[B9] Mahon GM, Wang Y, Korus M, Kostenko E, Cheng L, Sun T, Arlinghaus RB, Whitehead IP (2003). The c-Myc Oncoprotein Interacts with Bcr. Curr Biol.

[B10] Boguski MS, McCormick F (1993). Proteins regulating Ras and its relatives. Nature.

[B11] Van Aelst L, C DSS (1997). Rho GTPases and signaling networks. Genes Dev.

[B12] Lu W, Mayer BJ (1999). Mechanism of activation of Pak1 kinase by membrane localization. Oncogene.

[B13] Scita G, Tenca P, Frittoli E, Tocchetti A, Innocenti M, Giardina G, Di Fiore PP (2000). Signaling from Ras to Rac and beyond: not just a matter of GEFs. Embo J.

[B14] Radziwill G, Erdmann RA, Margelisch U, Moelling K (2003). The Bcr kinase downregulates Ras signaling by phosphorylating AF-6 and binding to its PDZ domain. Mol Cell Biol.

[B15] Whitehead IP, Campbell S, Rossman KL, Der CJ (1997). Dbl family proteins. Biochim Biophys Acta.

[B16] Alberts AS, Treisman R (1998). Activation of RhoA and SAPK/JNK signalling pathways by the RhoA-specific exchange factor mNET1. Embo J.

[B17] Grignani F, Kinsella T, Mencarelli A, Valtieri M, Riganelli D, Grignani F, Lanfrancone L, Peschle C, Nolan GP, Pelicci PG (1998). High-efficiency gene transfer and selection of human hematopoietic progenitor cells with a hybrid EBV/retroviral vector expressing the green fluorescence protein. Cancer Res.

[B18] Maru Y, Witte ON, Shibuya M (1996). BCR-ABL induces neurite-like structures and BCR lacking the SH2-binding domain induces cell rounding in PC12 cells. Exp Cell Res.

[B19] Bug G, Rossmanith T, Henschler R, Kunz-Schughart LA, Schroder B, Kampfmann M, Kreutz M, Hoelzer D, Ottmann OG (2002). Rho family small GTPases control migration of hematopoietic progenitor cells into multicellular spheroids of bone marrow stroma cells. J Leukoc Biol.

[B20] van Buul JD, Hordijk PL (2004). Signaling in leukocyte transendothelial migration. Arterioscler Thromb Vasc Biol.

[B21] Pfeifer H, Wassmann B, Hofmann WK, Komor M, Scheuring U, Bruck P, Binckebanck A, Schleyer E, Gokbuget N, Wolff T, Lubbert M, Leimer L, Gschaidmeier H, Hoelzer D, Ottmann OG (2003). Risk and prognosis of central nervous system leukemia in patients with Philadelphia chromosome-positive acute leukemias treated with imatinib mesylate. Clin Cancer Res.

[B22] Mohle R, Moore MA, Nachman RL, Rafii S (1997). Transendothelial migration of CD34+ and mature hematopoietic cells: an in vitro study using a human bone marrow endothelial cell line. Blood.

[B23] Chuang TH, Xu X, Kaartinen V, Heisterkamp N, Groffen J, Bokoch GM (1995). Abr and Bcr are multifunctional regulators of the Rho GTP-binding protein family. Proc Natl Acad Sci U S A.

[B24] Harnois T, Constantin B, Rioux A, Grenioux E, Kitzis A, Bourmeyster N (2003). Differential interaction and activation of Rho family GTPases by p210bcr-abl and p190bcr-abl. Oncogene.

[B25] Salesse S, Verfaillie CM (2002). Mechanisms underlying abnormal trafficking and expansion of malignant progenitors in CML: BCR/ABL-induced defects in integrin function in CML. Oncogene.

[B26] Unwin RD, Sternberg DW, Lu Y, Pierce A, Gilliland DG, Whetton AD (2005). Global effects of BCR/ABL and TEL/PDGFRbeta expression on the proteome and phosphoproteome: identification of the Rho pathway as a target of BCR/ABL. J Biol Chem.

[B27] Pelletier SD, Hong DS, Hu Y, Liu Y, Li S (2004). Lack of the adhesion molecules P-selectin and intercellular adhesion molecule-1 accelerate the development of BCR/ABL-induced chronic myeloid leukemia-like myeloproliferative disease in mice. Blood.

